# Semi-synthetic ocotillol analogues as selective ABCB1-mediated drug resistance reversal agents

**DOI:** 10.18632/oncotarget.4493

**Published:** 2015-07-04

**Authors:** Yun-Kai Zhang, Hengyuan Zhang, Guan-Nan Zhang, Yi-Jun Wang, Rishil J. Kathawala, Rui Si, Bhargav A. Patel, Jinyi Xu, Zhe-Sheng Chen

**Affiliations:** ^1^ Department of Pharmaceutical Sciences, College of Pharmacy and Health Sciences, St. John's University, Queens, NY 11439, USA; ^2^ Department of Medicinal Chemistry and State Key Laboratory of Natural Medicines, China Pharmaceutical University, Nanjing 210009, PR China

**Keywords:** ABC transporter, multidrug resistance, ABCB1, ginsenoside, ocotillol-type triterpenoid derivatives

## Abstract

Overexpression of ATP-Binding Cassette transporters leads to multidrug resistance in cancer cells and results in the failure of chemotherapy. In this *in-vitro* study, we investigated whether or not (20*S*, 24*R/S*)-epoxy-12β, 25-dihydroxy-dommarane-3β-amine (ORA and OSA), a pair of semi-synthetic ocotillol analogue epimers, could inhibit the ABCB1 transporter. ORA (1 μM and 3 μM) significantly reversed the resistance to paclitaxel and vincristine in ABCB1-overexpressing SW620/Ad300 and HEK/ABCB1 cells, whereas OSA had no significant effects. In addition, ORA (3 μM) significantly increased the intracellular accumulation of [^3^H]-paclitaxel by suppressing the efflux function of ABCB1. Meanwhile, both ORA (3 μM) and OSA (3 μM) did not significantly alter the expression level or the subcellular location of ABCB1 protein. Moreover, the ABCB1 ATPase study suggested that ORA had a stronger stimulatory effect on the ATPase activity than OSA. ORA also exhibited a higher docking score as compared with OSA inside transmembrane domain of ABCB1. Overall, we concluded that ORA reverse ABCB1-mediated MDR by competitively inhibiting the ABCB1 drug efflux function.

## INTRODUCTION

Multi-drug resistance (MDR) is the condition that enables cancerous cells to resist antineoplastic compounds with a wide variety of chemical structures and mechanisms, and therefore is a major cause of chemotherapy failure [1]. The most prominent factor for MDR occurring in cancer cells is the increased efflux of drugs [2, 3]. ATP-binding cassette (ABC) transporters utilize the energy generated by ATP hydrolysis to transport various substrates across cellular membranes, therefore contributing to elevated drug-efflux effect in cancer cells [4, 5]. In clinic, the ABC transporters frequently involved in MDR mainly include ABC subfamily B member 1 (ABCB1/P-glycoprotein/MDR1), multi-drug resistance protein 1(ABCC1/MRP1), and breast cancer resistance protein (ABCG2/BCRP/MXR) [5–7]. Substrates of ABCB1 include anthracyclines, topoisomerase inhibitors, vinca alkaloids, and taxanes [8–10]. Therefore, inhibition of ABCB1 gained research focus in order to resensitize MDR cancer cells to chemotherapeutical agents. Verapamil and cyclosporin A are classified as first-generation ABCB1 inhibitors [11, 12], while their derivatives such as PSC-833 are mostly classified as second-generation [13]. The third-generation includes inhibitors such as LY335979, VX-710, and XR-9576 [14]. However, these compounds either have severe side effects or alter the pharmacokinetic profile of anticancer drugs, so their application is impeded [15, 16]. Therefore, discovery of a novel lead compound as ABCB1 inhibitor is still urgent and promising for overcoming MDR in cancer chemotherapy.

**Figure 1:**
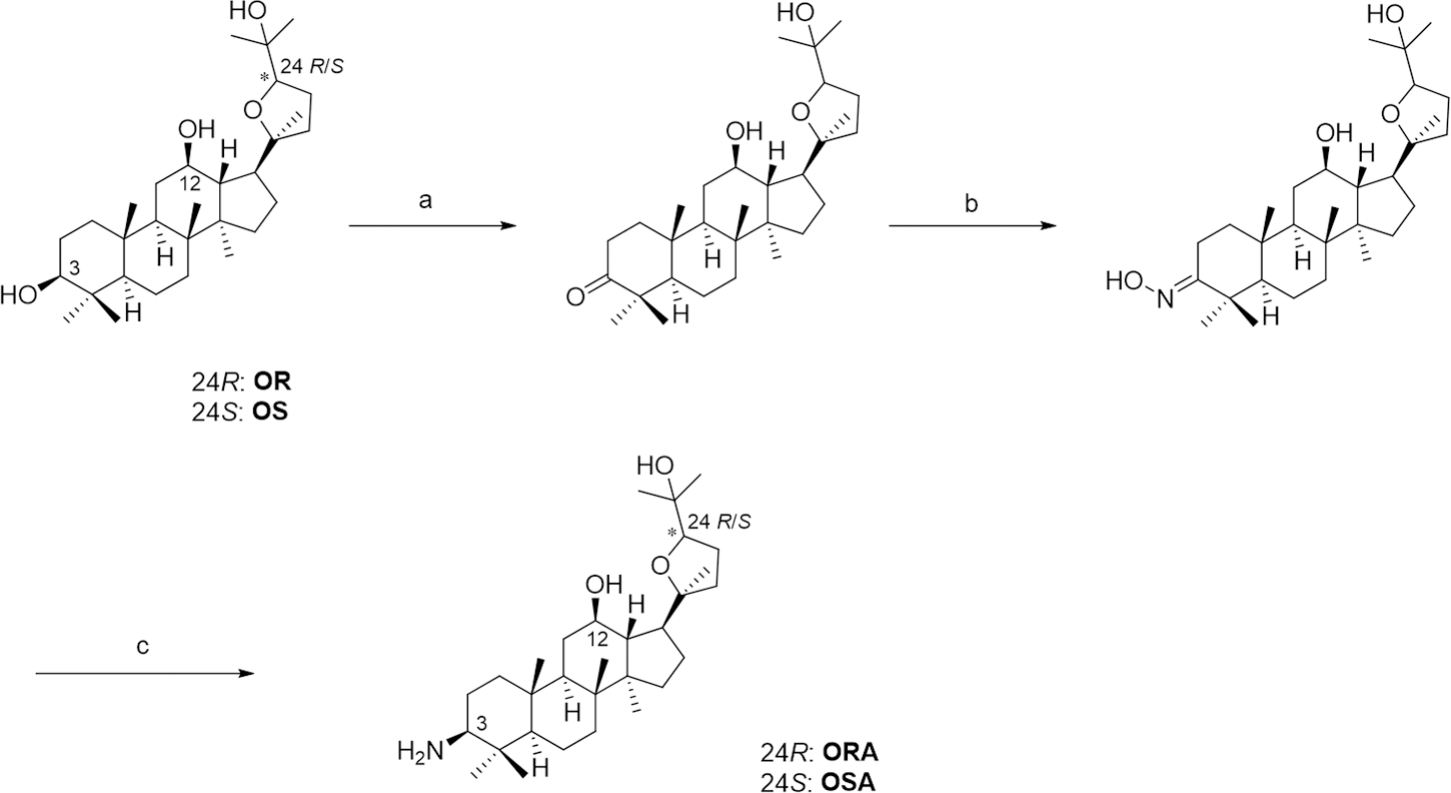
Synthesis of ORA and OSA Reagents and conditions: **A.** PCC, CH_2_Cl_2_, rt, 3 h **B.** Hydroxylamine hydrochloride, pyridine; 80°C, 2 h **C.** NaCNBH_3_, TiCl_3_, AcONH_4_, CH_3_OH, rt, 8 h.

Modification of natural active ingredients is one of the promising methods to identify new inhibitors for ABC transporters. Previously, several ginsenosides have been reported to have reversal effects on MDR cells [17]. Ginsenoside Rg3 has been observed to compete with anticancer drugs for binding to ABCB1, thereby competitively inhibits drug efflux [18]. Derived from Rg3, 20(*S*)-protopanaxadiol (aPPD) and its analogues have also been reported to show reversal nature of ABCB1 inhibition [19, 20]. Most ginsenosides share a dammarane-type triterpenoid saponin structure, while some minor ginsenosides isolated from *P. quinquefolius* with a modified steroid skeleton belong to ocotillol-type triterpenoids, of which the carbon chain at 20-position is replaced by a tetrahydrofuran ring [21]. To date few studies have been devoted to the MDR reversal effect of ocotillol-type triterpenoid derivatives. Therefore, we designed and tested a series of ocotillol-type analogues by modifying side chains of aPPD, aiming to acquire new ABC transporter inhibitors and to better understand their mechanisms. Our preliminary screening results (Supplemental Table 1) showed that the two 3-amino derivatives (20*S*, 24*R/S*)-epoxy-12β, 25-dihydroxy-dommarane-3β-amine (ORA and OSA, Figure 1) had the most potential MDR reversal activity. Herein, we report for the first time that ORA and OSA selectively reverse ABCB1-mediated MDR. The purpose of the present study was to further demonstrate the MDR reversal activities of ORA and OSA and elucidate their potential mechanisms.

## RESULTS

### ORA and OSA sensitized ABCB1-overexpressing cells to chemotherapeutic drugs

**Figure 2:**
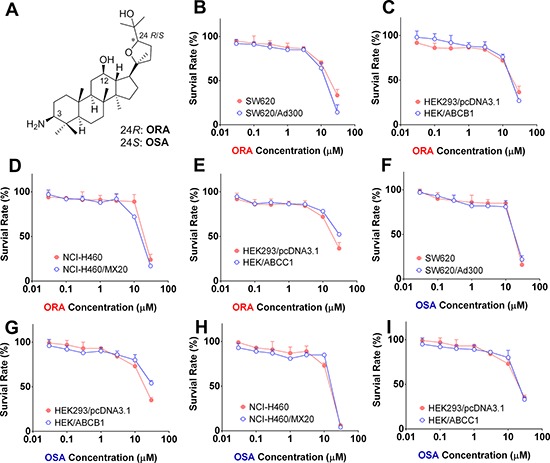
Cytotoxicity of ORA and OSA in MDR and parental cell lines **A.** Chemical structures of ORA and OSA. MTT assay was used to evaluate cytotoxicity of ORA or OSA in pairs of MDR and parental cell lines: SW620 and SW620/Ad300 **B** and **F.** HEK293/pcDNA3.1 and HEK/ABCB1 **C** and **G.** HEK293/pcDNA3.1 and HEK/ABCC1 **D** and **H.** NCI-H460 and NCI-H460/MX20 **E** and **I.** Representative curves were shown as cell survival rate verses concentration of compounds. Error bars represent the SD.

In order to investigate the effects of ORA and OSA on ABC transporters, we first examined the sensitivity of ABCB1-, ABCG2-, and ABCC1-overexpressing cells to ORA and OSA. Based on the results from the MTT assay, we determined that ORA and OSA could be considered as non-toxic (survival rate higher than 85%) up to 3 μM against any of the cell lines used in this study (Figure 2).

Next, we further examined whether ORA and OSA could increase the sensitivity of ABCB1-expressing drug-resistant cells to substrate drugs. As shown in Table 1, the ABCB1-overexpressing human colon cancer cells SW620/Ad300 showed much higher IC_50_ values to ABCB1 substrates paclitaxel and vincristine than parental SW620 cells did. Our results showed ORA at both 1 and 3 μM were able to significantly increase the sensitivity of SW620/Ad300 cells to paclitaxel and vincristine, and its efficacy showed a concentration-dependent pattern. OSA was also able to increase the sensitivity of SW620/Ad300 cells at 3 μM with a weaker efficacy than ORA. ORA, OSA, and verapamil did not alter the cytotoxicity of these substrate drugs in parental SW620 cells. The parental SW620 cells and drug resistant SW620/Ad300 cells exhibited similar sensitivity to cisplatin, which is not a substrate of ABCB1. The IC_50_ values of SW620 and SW620/Ad300 cell lines to cisplatin were also unaffected by ORA, OSA and verapamil. In the ABCB1-transfected HEK/ABCB1 and parental HEK293/pcDNA3.1 cell lines, a similar phenomenon was observed as shown in Table 2. ORA could significantly inhibit ABCB1-mediated drug resistance with a concentration-dependent pattern in HEK/ABCB1. OSA treatment at 3 μM also increased the sensitivity to ABCB1 substrates in HEK/ABCB1 cell line; however this reversal result did not yield a significant difference when compared to the control.

**Table 1:** Reversal effects of ORA and OSA to ABCB1-mediated MDR in parental and ABCB1-overexpressing cell lines

Treatment	IC_50_ value ± SD^a^ (nM, Resistance Fold^b^)	
	SW620	SW620/Ad300
Paclitaxel	2.13 ± 0.37 (1.00)	2141.59 ± 241.34 (1005.44)
+ OSA 3 μM	2.21 ± 0.16 (1.04)	1509.43 ± 181.51 (708.65)*
+ ORA 1 μM	2.33 ± 0.10 (1.09)	186.10 ± 11.91 (87.37)*
+ ORA 3 μM	1.91 ± 0.11 (0.90)	82.36 ± 3.87 (38.67)*
+ Verapamil 3 μM	1.76 ± 0.22 (0.83)	28.44 ± 3.01 (13.35)*
		
Vincristine	8.43 ± 0.91 (1.00)	2215.91 ± 156.64 (257.66)
+ OSA 3 μM	7.17 ± 0.34 (0.84)	1224.30 ± 106.51 (142.36)*
+ ORA 1 μM	6.92 ± 0.84 (0.81)	281.05 ± 23.89 (32.68)*
+ ORA 3 μM	7.41 ± 0.49 (0.86)	76.81 ± 4.38 (8.93)*
+ Verapamil 3 μM	9.33 ± 0.45 (1.08)	39.94 ± 1.72 (4.64)*
		
Cisplatin	719.40 ± 68.46(1.00)	842.75 ± 77.66(1.17)
+ OSA 3 μM	788.85 ± 69.18(1.10)	982.95 ± 80.42(1.37)
+ ORA 1 μM	960.31 ± 84.88(1.33)	960.94 ± 69.96(1.34)
+ ORA 3 μM	732.74 ± 93.15(1.02)	999.98 ± 108.14(1.39)
+ Verapamil 3 μM	786.34 ± 63.41(1.09)	841.14 ± 79.01(1.17)

a IC_50_ values are represented as mean ± SD of three independent experiments performed in triplicate.

b Resistance fold was calculated by dividing the IC_50_ values of substrates in the presence or absence of inhibitor by the IC_50_ of parental cells without inhibitor.

* *p* < 0.05 versus control group

**Table 2:** Reversal effects of ORA and OSA to ABCB1-mediated MDR in parental and ABCB1-transfected cell lines

Treatment	IC_50_ value ± SD^a^ (nM, Resistance Fold^b^)	
	HEK293/pcDNA3.1	HEK/ABCB1
Paclitaxel	146.51 ± 13.67 (1.00)	8990.19 ± 617.31 (61.36)
+ OSA 3 μM	138.28 ± 6.22 (0.94)	7769.45 ± 744.28 (53.03)
+ ORA 1 μM	174.76 ± 12.56 (1.18)	1954.96 ± 56.30 (13.34)*
+ ORA 3 μM	158.78 ± 12.87 (1.08)	689.71 ± 57.24 (4.71)*
+ Verapamil 3 μM	142.62 ± 6.46 (0.97)	310.69 ± 28.73 (2.12)*
		
Vincristine	52.21 ± 7.93 (1.00)	1598.26 ± 182.12 (30.61)
+ OSA 3 μM	51.33 ± 2.92 (0.98)	1117.13 ± 137.42 (21.40)
+ ORA 1 μM	50.03 ± 1.49 (0.96)	752.58 ± 15.12 (14.41)*
+ ORA 3 μM	42.69 ± 4.32 (0.81)	286.14 ± 8.15 (5.48)*
+ Verapamil 3 μM	55.05 ± 2.55 (1.05)	130.85 ± 4.33 (2.51)*
		
Cisplatin	652.72 ± 86.35(1.00)	996.34 ± 107.42(1.53)
+ OSA 3 μM	694.52 ± 86.73(1.06)	868.50 ± 111.28(1.33)
+ ORA 1 μM	790.54 ± 73.04(1.21)	819.38 ± 68.26(1.25)
+ ORA 3 μM	646.73 ± 71.52(0.99)	792.77 ± 83.67(1.21)
+ Verapamil 3 μM	776.84 ± 70.81(1.19)	934.84 ± 88.54(1.43)

a IC_50_ values are represented as mean ± SD of three independent experiments performed in triplicate.

b Resistance fold was calculated by dividing the IC_50_ values of substrates in the presence or absence of inhibitor by the IC_50_ of parental cells without inhibitor.

* *p* < 0.05 versus control group

As shown in Table 3, ORA and OSA at 3 μM did not affect ABCG2- and ABCC1-mediated MDR significantly when compared with fumitremorgin C (FTC) and PAK-104P as positive controls.

**Table 3:** Effects of ORA and OSA in parental and ABCG2- and ABCC1-mediated MDR cell lines

**Treatment**	**IC_50_ value ± SD^a^ (nM, Resistance Fold)**	
	**NCI-H460**	**NCI-H460/MX20**
Mitoxantrone	55.91 ± 6.38 (1.00)	3287.17 ± 517.65 (58.79)
+ OSA 3 μM	50.39 ± 3.41 (0.90)	2633.79 ± 270.86 (47.11)
+ ORA 3 μM	46.81 ± 5.51 (0.84)	2543.22 ± 208.20 (45.48)
+ FTC 3 μM	48.14 ± 4.28 (0.86)	104.70 ± 10.69 (1.86)*
**Treatment**	**IC_50_ value ± SD^a^ (nM, Resistance Fold)**	
	**HEK293/pcDNA3.1**	**HEK293/ABCC1**
Vincristine	62.35 ± 9.76 (1.00)	732.32 ± 78.16 (11.75)
+ OSA 3 μM	61.05 ± 8.54 (0.97)	781.23 ± 102.26 (12.53)
+ ORA 3 μM	52.32 ± 8.81 (0.84)	751.41 ± 92.93 (12.05)
+ PAK-104P 10 μM	49.18 ± 6.76 (0.79)	90.65 ± 13.22 (1.45)*

a IC_50_ values are represented as mean ± SD of three independent experiments performed in triplicate.

b Resistance fold was calculated by dividing the IC_50_ values of substrates in the presence or absence of inhibitor by the IC_50_ of parental cells without inhibitor.

* *p* < 0.05 versus control group

Based on the above results, it appeared that ORA significantly and selectively inhibited ABCB1-mediated MDR in both SW620/Ad300 and HEK/ABCB1 cells, while OSA exhibited reversal effect in SW620/Ad300 cells but not in HEK/ABCB1 cells.

### ORA increased the accumulation of [^3^H]-paclitaxel in ABCB1-overexpressing cells

**Figure 3:**
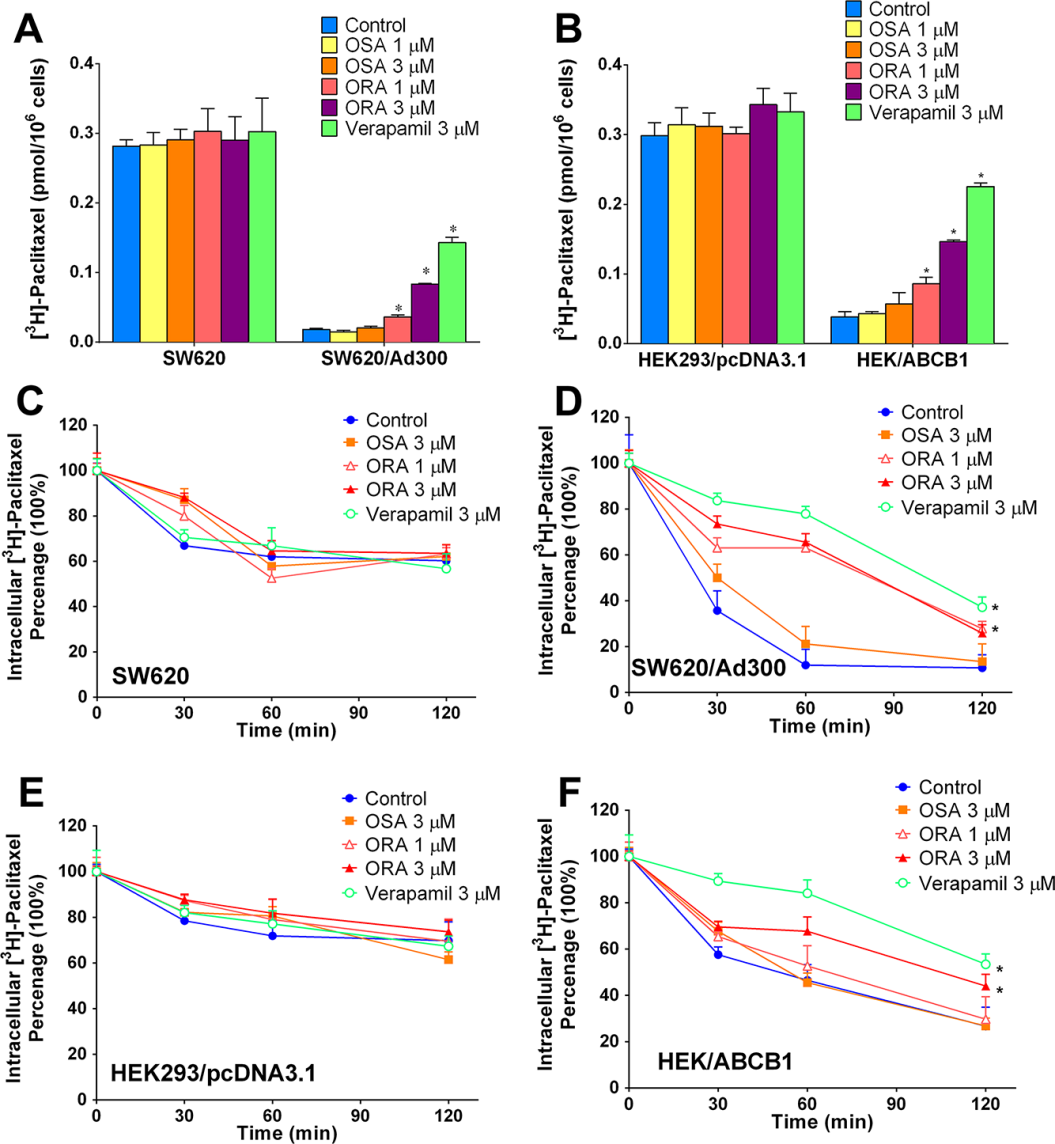
Effect of ORA or OSA on accumulation and efflux of [^3^H]-paclitaxel Effect of ORA or OSA on accumulation of [^3^H]-paclitaxel in **A.** SW620 and SW620/Ad300 cells, **B.** HEK293/pcDNA3.1 and HEK/ABCB1 cells. A time course verses percentage of intracellular [^3^H]-paclitaxel remaining was plotted (0, 30, 60, 120 min) to show effects of ORA or OSA in **C.** SW620 and **D.** SW620/Ad300 cells, **E.** HEK293/pcDNA3.1 and **F.** HEK/ABCB1 cells. *p < 0.05 versus the control group. Error bars represent the SD. Experiments were performed at least three independent times.

To further investigate the potential mechanism by which ORA and OSA sensitized ABCB1-overexpressing cells to anticancer drugs, we determined the intracellular drug accumulation level by radioactive-labeled ABCB1 substrate [^3^H]-paclitaxel. As shown in Figure 3A and Figure 3B, intracellular [^3^H]-paclitaxel levels were significantly lower in ABCB1-overexpressing SW620/Ad300 and HEK/ABCB1 cells than those in parental cells. Verapamil at same concentration in MTT assay (3 μM) was used as the positive control group. ORA, OSA and verapamil did not alter the intracellular paclitaxel accumulation significantly in parental SW620 and HEK293/pcDNA3.1 cells. ORA at 1 and 3 μM significantly increased accumulation of [^3^H]-paclitaxel in ABCB1-overexpressing SW620/Ad300 cells (Figure 3A) and similar results were observed in transfected HEK/ABCB1 cells (Figure 3B). These data showed consistent trend with the MTT assays, and therefore suggested that ORA sensitized ABCB1-overexpressing cells to anticancer drugs by increasing their intracellular accumulation.

### ORA inhibited the efflux of [^3^H]-paclitaxel in ABCB1-overexpressing cells

ABC transporters were known to mediate multi-drug resistance by actively pumping out anti-cancer drugs therefore lowering their intracellular concentration [22]. Therefore, in order to further determine the mechanism of drug accumulation, we performed a time-course efflux assay using tritium-labeled [^3^H]-paclitaxel to determine ABCB1-mediated drug efflux. As shown in Figure 3C–3F, the intracellular [^3^H]-paclitaxel in ABCB1-overexpressing SW620/Ad300 and HEK/ABCB1 cells decreased much faster over time than those in the parental cells, suggesting an active process of drug efflux mediated by ABCB1. As shown in Figure 3C and 3E, ORA, OSA and verapamil did not significantly influence paclitaxel efflux in parental SW620 and HEK293/pcDNA3.1 cells. However, ORA at 3 μM significantly inhibited the efflux function of ABCB1 (Figure 3D and 3F).

### ORA and OSA had no effects on the expression of ABCB1 in SW620/Ad300 cells

**Figure 4:**
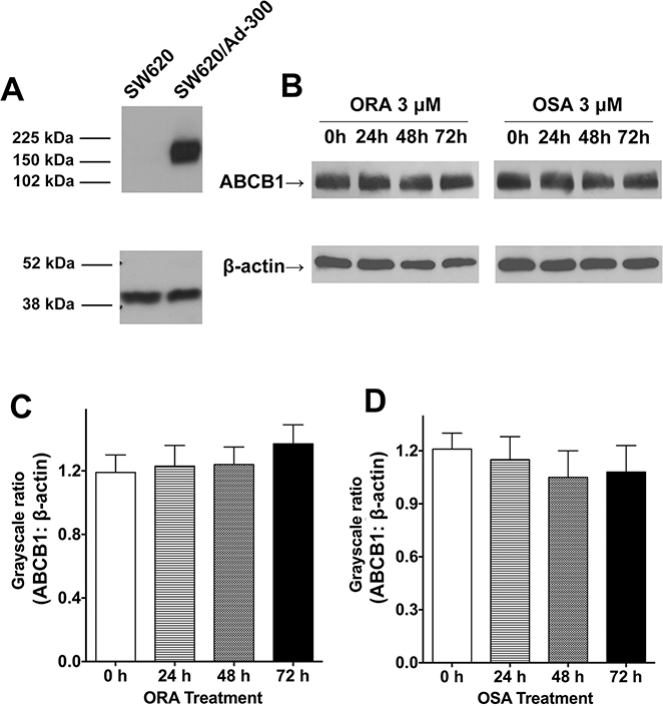
Western blot analysis of ABCB1, the effect of ORA or OSA on the expression levels of ABCB1 transporter **A.** The expression of ABCB1 in SW620 and SW620/Ad300 cell lysates. **B.** The effect of ORA or OSA at 3 μM on the expression levels of ABCB1 in SW620/Ad300 cells for 24, 36 and 72 h. Quantitative analysis of effects of **C.** ORA or **D.** OSA on ABCB1 expression. Equal amounts of total cell lysate were used for each sample. Representative result was shown here and similar results were obtained in other trials.

In order to investigate whether ORA and OSA can alter the expression pattern of ABCB1, we performed a western blot assay for ABCB1 in parental SW620 and drug-resistant SW620/Ad300 cells. As shown in Figure 4A, SW620/Ad300 over-expressed ABCB1 transporter therefore showing a band at about 170 kDa, whereas parental SW620 cells have no band at same position. As shown in Figure 4B–4D, the treatment of ORA and OSA at 3 μM did not significantly change the expression level of ABCB1 in SW620/Ad300 cells.

### ORA and OSA did not alter the subcellular localization of ABCB1 in ABCB1-overexpressing cells

**Figure 5:**
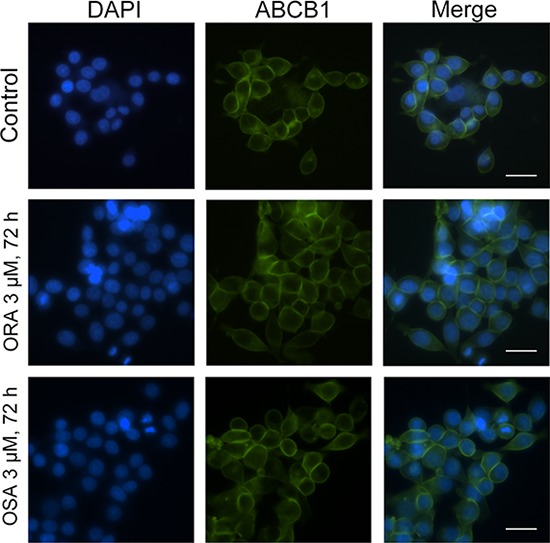
Effect of ORA and OSA on the subcellular localization of ABCB1 Subcellular localization of ABCB1 in ABCB1-overexpressed SW620/Ad300 cells (top panel). Effect of ORA at 3 μM (middle panel) and OSA at 3 μM (bottom panel) on the subcellular localization of ABCB1. Scale bar, 20 μm, DAPI (blue) counterstains the nuclei.

In order to demonstrate whether ORA or OSA can influence subcellular localization of ABCB1 transporter, we performed an immune-fluorescence assay in ABCB1 overexpressing cells. As shown by the FITC-conjugated antibody, ABCB1 transporters showed a membrane-located expression pattern in SW620/Ad300 cells (Figure 5), and the incubation of these cells with 3 μM of ORA or OSA for 72 h did not significantly alter the subcellular distribution pattern of ABCB1 when compared to control.

### ORA and OSA stimulated ATPase activity of ABCB1

**Figure 6:**
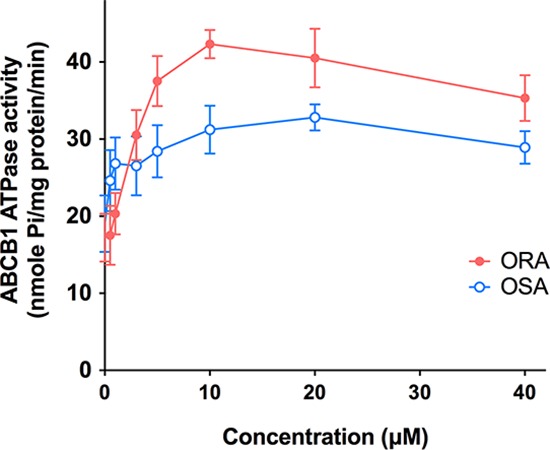
Effect of ORA and OSA on the Vi-sensitive ABCB1 ATPase activity Crude membranes (100 μg protein/ml) from High-five cells expressing ABCB1 were incubated with increasing concentrations of ORA and OSA (0–40 μM), in the presence and absence of sodium orthovanadate (Vi) (0.3 mM), in ATPase assay buffer as described in Section 2.7

ABCB1 transporter utilizes energy derived from the hydrolysis of ATP to efflux their substrates across the membrane against a concentration gradient; thus, ATP consumption reflects its ATPase activity. To assess the effect of ORA and OSA on the ATPase activity of ABCB1, we measured ABCB1-mediated ATP hydrolysis in the presence of ORA or OSA at various concentrations from 0 to 40 μM. Interestingly, ORA stimulated the ATPase activity of ABCB1 in a concentration-dependent manner, with a maximal stimulation of 2.46-fold of the basal activity (Figure 6). In contrast, the maximal stimulation of OSA is 1.78-fold of the basal activity. The Figure 6 demonstrates that the concentration of ORA required to obtain 50% stimulation is 2.08 μM.

### Docking analysis of ORA and OSA within human ABCB1 homology model and human CYP3A4

**Figure 7:**
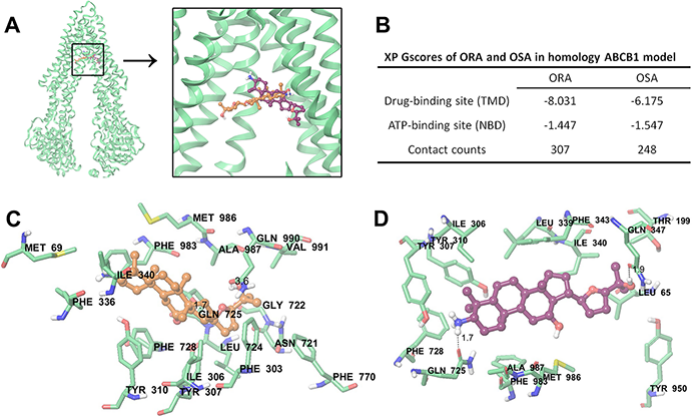
Molecular modeling of binding of ORA or OSA to homology ABCB1 **A.** Location of ORA (orange) and OSA (purple) molecules in the ABCB1 internal cavity. TM9, 10 and 12 are omitted for better view. **B.** Glide XP GScores for ORA or OSA at different sites of ABCB1. **C.** The docked conformation of ORA within the binding cavity of ABCB1 is shown as a ball and stick model. Important residues are depicted as sticks with the atoms colored as follows: carbon, green; hydrogen, white; nitrogen, blue; oxygen, red; sulfur, yellow; whereas ORA is shown with the same color scheme as above except the carbon atoms are presented in orange. The dotted black lines indicate hydrogen-bonding interactions. **D.** The docked conformation of OSA. Color scheme is same as panel (C) except carbon atoms of OSA are presented in purple.

The existing results have indicated that ORA and OSA may have direct interactions with ABCB1 transporter. Therefore, we performed a molecular docking simulation to confirm our hypothesis. ORA and OSA were more preferable to bind at the transmembrane domain (TMD) instead of intracellular nuclear binding domain (NBD) in ABCB1 (Figure 7B). However their binding poses did not completely overlap, as shown in Figure 7A.

The docked pose of ORA into the large drug-binding cavity of human ABCB1 is shown in Figure 7C. The triterpene core of ORA, 20, 24-expoxydammarane was stabilized into a large cavity formed by hydrophobic residues Met69, Phe303, Ile306, Tyr307, Tyr310, Phe336, Leu339, Ile340, Phe343, Leu724, Phe728, Phe983, Met986, Ala987, and Val991. The 3-ionized NH_3_^+^ group formed cation-π interaction with the phenyl ring of Phe336. The 12′-OH group interacted with side chain carbonyl group of Gln725 to form a hydrogen bond (−OH···OC-Gln725, 1.7 Å). 25′-OH group formed another hydrogen bond with carbonyl oxygen of Gln990 (−OH···OC-Gln990, 3.6 Å).

The docked pose of OSA is shown in Figure 7D. The 20, 24-epoxydammarane core was stabilized by hydrophobic residues Leu65, Ile306, Tyr307, Tyr310, Leu339, Ile340, Phe343, Phe728, Phe983, Met986, and Ala987. The 25′-OH group formed hydrogen bond with carbonyl group of Gln347 (−OH···OC-Gln347, 1.9 Å). Similarly, 3′-ionized amino group formed hydrogen-bonding interaction with side chain carbonyl group of Gln725 (−NH_3_···OC-Gln725, 1.7 Å). Moreover, NH_3_^+^ group formed cation-π interaction with Tyr307.

We also performed molecular docking of at human CYP3A4. Paclitaxel, as substrate of CYP3A4, exhibited a Glide score of −13.411. Biricodar (VX-710) is a second generation ABCB1 inhibitor that is known to inhibit CYP3A4 and then decrease the clearance of paclitaxel [23]. Biricodar exhibited a Glide score of −10.841. Redocking of the native bound ritonavir analogue had a Glide score of −11.249. However, our compounds ORA had score of −8.446 while OSA had a score of −8.522 followed same docking protocols.

## DISCUSSION

Natural environment is a fertile ground for discovery of novel lead compounds for the development of new drugs. In this study, for the first time we demonstrate that our natural sourced semi-synthesized compound ORA, a derivative from 20(*S*)-protopanaxadiol, significantly reduced the resistance of MDR cells to ABCB1 substrates, while its epimer OSA had non-significant effects.

First, we examined the toxic effects of ORA and OSA alone on all the cell lines we utilized (Figure 2) and determined their IC_15_ values (Supplemental Table 2). Based on these data, the concentrations we used for the following studies (1 μM and 3 μM) were regarded as non-cytotoxic. Therefore, ORA and OSA sensitized the MDR cells through altering cell function instead of killing cells together with anti-neoplastic agents.

Originally, ABCB1-overexpressing SW620/Ad300 cells had 1005- and 258-times resistance to paclitaxel and vincristine respectively as compared to that of parental SW620 cells. Pre-incubation with 3 μM OSA only slightly sensitized SW620/Ad300 to substrates, whereas its 24(*R*)-epimer ORA at 1 and 3 μM significantly reduced resistance in the MDR cells in a concentration-dependent manner (Table 1). This ORA-induced change in resistance was directly related to ABCB1 as follows: (i) ORA did not produce any significant toxic effect on either parental or drug-selected cells. (ii) ORA did not potentiate effects of paclitaxel and vincristine on parental SW620 cells, which did not express ABCB1. (iii) ORA did not significantly change IC_50_ values of cisplatin, which is not an ABCB1 substrate. Verapamil (3 μM), a known ABCB1 inhibitor, also exhibited similar effects, however cisplatin did not. In order to further examine their specificity, we also determined the effects of ORA and OSA in ABCB1-transfected cell lines. Similarly, the results showed the same trend as those in the drug-selected cell lines and therefore, supported our hypothesis that ORA suppresses ABCB1 transporter to reverse MDR. Collectively, the evidence suggests that ORA can specifically inhibit ABCB1 function in both drug-selected SW620/Ad300 cells and transfected HEK/ABCB1 cells.

As reported, ABCG2 and ABCC1 transporters also lead to cancer MDR. Therefore, we evaluated the effects of ORA and OSA on ABCG2- and ABCC1- overexpressing MDR cells. As a result of overexpression of ABCG2, NCI-H460/MX20 were resistant to mitoxantrone as compared to parental NCI-H460 cells, and the resistance could be significantly reversed by 3 μM FTC. Our results reveal OSA and ORA at 3 μM had weak or no effect on ABCG2 as they failed to alter IC_50_ values in both parental and MDR cell lines (Table 2). Likewise, ORA and OSA at 3 μM could not reverse ABCC1-mediated MDR as PAK-104P at 10 μM did. Altogether, our major finding is that ORA would selectively reverse ABCB1-mediated MDR in a concentration-dependent manner without any effects on ABCG2- and ABCC1-mediated MDR.

In order to further understand the mechanism of ORA's reversal effect, we conducted a radiolabeled drug-accumulation study to evaluate the intracellular level of [^3^H]-paclitaxel. Our results showed that MDR cells had lower levels of intracellular [^3^H]-paclitaxel accumulation than parental cells had and, therefore, were less sensitive. ORA sensitized MDR cells by significantly increasing the intracellular levels of paclitaxel. We subsequently performed an efflux assay to determine whether the accumulation effect was directly associated with inhibition of the ABCB1 function or due to alternation in uptake of the drug. Our results showed that ABCB1-mediated drug efflux was significantly suppressed by ORA, leading to significant retention of intracellular drug in SW620/Ad300 and HEK/ABCB1 cells.

Previously it has been suggested that suppressed ABC transporter-mediated efflux could result from protein down-regulation [24]. However, our Western blot analysis showed that there was no decrease in ABCB1 protein level after treatment. Moreover, as we performed Western blotting using total cell lysates, we further conducted an immune-fluorescence study in SW620/Ad300 cells to determine the subcellular location of ABCB1. ABCB1 showed a membrane-like localization pattern in ABCB1-overexpressing cells [25]. Our results showed that there are no changes in the subcellular location of ABCB1 after treatment of ORA and OSA as compared to untreated control. These findings indicated that ORA might directly inhibit ABCB1 other than down-regulate or translocate ABCB1 protein.

As previously reported, the model of substrate transport by ABCB1 can be summarized in two stages. Firstly, the substrate enters into the internal drug-binding pocket through an open portal. Secondly, ATP-binding at the NBDs causes a large conformational change, presenting the substrate and the drug-binding sites to the outer leaflet [9]. In that case, an ATP modulator could either bind to drug-binding pocket in TMD as a competitive inhibitor, or block ATP-binding in NBDs as a non-competitive inhibitor. It was interesting that ORA and OSA had different inhibitory efficacy as a pair of enantiomer. It can be concluded from out results that ORA had stronger effect on the ATPase activity of ABCB1 than OSA, by behaving as typical substrate and binding at the drug-substrate-binding site of ABCB1 transporter.

The molecular docking study further supports the results which indicate that ORA and OSA had direct interactions with ABCB1. Glide gscore is an empirical scoring function that approximates the ligand binding free energy [26]. The Glide gscores of both ORA and OSA at NBDs (ATP-binding sites) were much lower than those at TMDs as shown in Figure 7B. Therefore, ORA or OSA may not likely block ATP binding at NBDs, which is also supported by our ATPase results. An underlying correlation between ABCB1 inhibitory activity and lipophilicity of the compounds has already been suggested [27]. Hydrophobic compounds may distribute more into biomembrane from which ABCB1 extracts the substrates as previously illustrated [9]. ORA and OSA also exhibit the some pharmacophoric features such as hydrophobic groups, hydrogen bond acceptors, and positively charged ionizable group that have been described as critical for ABCB1 inhibition [28, 29]. However, although ORA and OSA shared these features, the gscores expressed in kcal/mol for ORA and OSA were found to be (−8.031) and (−6.175) respectively. Weaker reversal effect of OSA could partially be explained by its poor binding energy score [29]. Moreover, OSA had a fewer contact counts in docking study when compared to that of ORA at their best docking pose respectively, which may also indicate its weaker interaction with ABCB1. Collectively, our docking results indicated the importance of 24*R*-conformer of 20, 24-epoxy-dammarane derivatives in ABCB1 inhibition and provided clues in molecular level for further optimization.

The safety of herb components is ensured by the continuous and long history of usage in large amounts as part of normal daily diet [30]. ORA and OSA are semi-synthetic derivatives of ocotillol-type triterpenoid obtained from ginseng. Previously, Li *et al* identified the formation of 20.24-epoxy-dammarane derivatives as main metabolite of aPPD in human liver microsomes and human hepatocytes [31]. Furthermore, an *in vivo* study described that ocotillol at 10 mg/kg did not have any toxic effects on nude mice in a period of 24 days [32]. Hence, these studies provided a great potential for *in vivo* usage of ORA. Furthermore, as compared with ligands that are known to inhibit CYP3A4, ORA and OSA have relatively lower docking scores. These molecular modelings predicted ORA and OSA have less potential to have strong interactions with CYP3A4. Overall, ORA and OSA are considered as ideal candidates for ABCB1 inhibitor with potential efficacy *in vivo*.

In conclusion, this study is the first to demonstrate that ORA reverses ABCB1-mediated MDR by blocking pump's efflux function. These results suggest that ORA may have the potential to be used in combination with conventional antineoplastic drugs (ABCB1 substrates) to augment or resensitize tumor chemotherapy.

## MATERIALS AND METHODS

### Chemicals

[^3^H]-paclitaxel (15 Ci/mmol) was purchased from Moravek Biochemicals, Inc (Brea CA). Dulbecco's modified Eagle's medium (DMEM), fetal bovine serum (FBS), penicillin/streptomycin and trypsin 0.25% were purchased from Hyclone (Thermo Scientific, Logan, UT). Monoclonal antibody C219 (against ABCB1) was purchased from EMD Millipore Corporation (Billerica, MA). Monoclonal antibodies sc-47778 (against β-actin) and secondary HRP-labeled rabbit anti-mouse IgG were purchased from Santa Cruz Biotechnology, Inc. (Dallas, TX). Monoclonal antibody P7965 (against ABCB1), paclitaxel, vincristine, cisplatin, mitoxantrone, verapamil, 3-(4, 5-dimethylthiazol-yl)-2, 5-diphenyltetrazolium bromide (MTT), dimethylsulfoxide (DMSO), Triton X-100, paraformaldehyde, ammonium molybdate, MES hydrate, antimony potassium tartrate, sodium azide and N-methyl-D-glucamine were obtained from Sigma Chemical Co. (St. Louis, MO). Potassium phosphate, EGTA and ATP were products of AMRESCO (Solon, OH). Sulfuric acid solution (37N) was purchased from Fisher Scientific (Pittsburgh, PA). KCl was a product of Avantor Performance Materials (Center Valley, PA). Ouabain was purchased from Enzo Life Sciences, Inc. (Farmingdale, NY). Dithiothreitol was a product of Promega Corporation (Madison, WI). MgCl_2_ was purchased from EMD Millipore (Billerica, MA). Ascorbic acid was a product of VWR International (West Chester, PA). Sodium orthovanadate was purchased from Alfa Aesar (Ward Hill, MA). PAK-104P was a gift of Prof. Shin-Ichi Akiyama (Kagoshima University, Kagoshima, Japan) from Nissan Chemical Ind. Co., Ltd. (Chiba, Japan). Fumitremorgin C (FTC) was a gift from Dr. Susan Bates (NIH, Bethesda, MD).

As previously described [33], ocotillol-type triterpenoidderivatives OR and OS were prepared from 20(*S*)-protopanaxadiol (PPD). After deriving 3-one (70% yield) with pyridiniumchlorochromate (PCC) in dichloromethane, it was reacted with hydroxylamine hydrochloride in pyridine to give the oxime in 90% yield. Then, the reduction of oxime with sodium cyanoborohydride in the presence of titanium(III) chloride and ammonium acetate in methanol afforded ORA and OSA, respectively (72% yield, Figure 1).

### Cell lines and cell culture

HEK293/pcDNA3.1, HEK/ABCB1 and HEK/ABCC1 cells were established by transfecting HEK293 with either the empty pcDNA3.1 or vector containing the full length *ABCB1* (HEK/ABCB1) or *ABCC1* (HEK/ABCC1), and were cultured in a medium containing 2 mg/ml of G418 [34]. The human colon cancer cell line SW620 and its doxorubicin-selected ABCB1-overexpressing SW620/Ad300 cell line were used for reversal study. Non-small cell lung cancer NCI-H460 cells and mitoxantrone-selected ABCG2-overexpressing NCI-H460/MX20 cells were kindly provided by Drs. Susan Bates and Robert Robey (NCI, NIH, Bethesda, MD). All the cell lines were grown as adherent monolayers in flasks with DMEM supplemented with 10% fetal bovine serum and 1% penicillin/streptomycin in a humidified incubator containing of 5% CO_2_ at 37°C.

### Cell cytotoxicity by MTT assay

The MTT colorimetric assay was used to detect the sensitivity of the cells against anticancer drugs. Cells (5 × 10^3^/well) were seeded evenly into (160 μl/well) 96-well plates and cultured overnight. For the reversal experiments, ORA, OSA, and parallel control modulators (20 μl/well) were added 1 h prior. Different concentrations of the chemotherapeutic drugs (20 μl/well) were then added into the designated wells. After 72 h of incubation, 20 μl of MTT solution (4 mg/ml) was added to each well, and the plate was further incubated for 4 h. Subsequently, the medium was discarded, and 100 μl of dimethylsulfoxide (DMSO) was added into each well to dissolve the formazan crystals. The absorbance was determined at 570 nm by the OPSYS microplate reader (DYNEX Technology, Inc., Chantilly, VA). The IC_50_ value was calculated from the survival curves using the modified Bliss method [35]. Verapamil was used at a nontoxic concentration of 3 μM as a positive control for ABCB1 overexpressing cell lines. FTC at 3 μM and PAK-104P at 10 μM were used as positive control for ABCG2 and ABCC1 overexpressing cell lines, respectively.

### [^3^H]-paclitaxel accumulation assay

The accumulation of [^3^H]-paclitaxel in HEK293/pcDNA3.1, HEK/ABCB1, SW620 and SW620/Ad300 cells was measured in the presence or absence of inhibitors. Cells were typsinized after they reached 80% confluence, then five aliquots from each cell line were suspended in the medium. Cells were then pre-incubated with PBS, ORA (1 μM and 3 μM, respectively), OSA (3 μM), or verapamil (3 μM) at 37°C for 1 h. Subsequently, cells were suspended in the medium containing 0.1 μM [^3^H]-paclitaxel for 2 h in the presence or absence of the reversal compounds at 37°C. After washing three times with ice cold PBS, the cells were lysed by adding lysis buffer (pH 7.4, containing 1% Triton X-100 and 0.2% SDS). Each sample was placed in 5 ml scintillation fluid and radioactivity was measured in the Packard TRI-CARB 1900CA liquid scintillation analyzer from Packard Instrument Company, Inc (Downers Grove, IL).

### [^3^H]-paclitaxel efflux assay

To measure the drug efflux, the cells were pretreated for 1 h with PBS, ORA at 1 μM and 3 μM, OSA at 3 μM, or verapamil at 3 μM. Radioactive substrate [^3^H]-paclitaxel was then added, and cells were further incubated for 2 h at 37°C. Cells were washed three times with ice-cold PBS, and then were supplemented with fresh medium with or without inhibitors. After 0, 30, 60 and 120 min, the aliquots of cells were removed and washed with ice-cold PBS immediately. Radioactivity was then measured as previously described [36].

### Preparation of total cell lysates

Cell extracts were prepared by incubating with lysis buffer (10 mM Tris HCl, pH 7.5, 1 mM EDTA, 0.1% SDS, 150 mM NaCl, 1% Triton X-100 and 0.01% leupeptin) for 20 min, followed by centrifugation at 12, 000 × g at 4°C for 20 min. The supernatant containing total cell lysates was stored at −80°C until gel electrophoresis was performed. Protein concentrations were determined by bicinchoninic acid (BCA™) based protein assay (Thermo Scientific, Rockford, IL).

### Western blotting analysis

Equal amounts of total cell lysate (20 μg protein) were resolved by sodium dodecyl sulfate polyacrylamide gel electrophoresis (SDS-PAGE) and electrophoretically transferred onto polyvinylidene fluoride (PVDF) membranes. The membranes were blocked and then immunoblotted with primary monoclonal antibodies against either actin at 1:400 dilution or ABCB1 at 1:100 dilution at room temperature for 2 h. They were further incubated for 1 h at room temperature with horseradish peroxide (HRP)-conjugated secondary antibody (1:1000 dilution). The protein-antibody complex was detected by enhanced chemiluminescence detection system (Amersham, NJ) as previously described [37]. The protein expression was quantified by ImageJ software (NIH, Bethesda, MD, USA).

### ABCB1 ATPase assay

The vanadate (Vi)-sensitive ATPase activity of ABCB1 in the membrane vesicles of High Five insect cells was measured as described previously [38]. The membrane vehicles were incubated in the ATPase assay buffer with or without vanadate, in different concentrations of ORA and OSA, and then in Mg-ATP in chronological order followed previously described protocols [39]. The reactions were stopped after 20 minutes and the liberated inorganic phosphate was measured as previously described [39].

### Immunofluorescence assay

SW620 and SW620/Ad300 cells were incubated with 3 μM of ORA, 3 μM of OSA or PBS buffer respectively for 72 h. The cells were then washed with PBS and fixed in 4% paraformaldehyde. Subsequently, cells were incubated with BSA (2 mg/ml) for 1 h followed by polyclonal antibody against ABCB1 for 2 h at 37°C. Cells were further incubated with FITC-conjugated anti-mouse IgG for 1 h. 2-(4-amidinophenyl)-6-indolecarbamidene dihydrochloride (DAPI) solution was applied to counterstain the nuclei. Images were taken with an inverted IX70 microscope (Olympus, Center Valley, PA) followed our previous protocol [40].

### Molecular modeling

ORA, OSA, paclitaxel, biricodar were built and prepared as ligands by our previous molecular modeling protocols [40]. The output files containing at most 100 unique conformers were used as input for docking simulations into human ABCB1 or human CYP3A4. The human ABCB1 homology model based on refined mouse ABCB1 was kindly provided by S. Aller and was used to generate docking grid [41]. Crystal structure of human CYP3A4 (PDB ID: 4K9W) was used for protein preparation. Protein Preparation Wizard (Epik version 2.5; Impact version 6.0, Schrödinger, LLC, New York, NY, 2013) default protocol was followed for protein refinement, in which the protonation states of residues were adjusted to the dominant ionic forms at physiological pH [42]. ABCB1 docking grid was generated by selecting all conserved drug interacting residues as centroid [41]. CYP3A4 grid was generated by selecting co-crystalized ligand. The co-crystalized ligand, a desoxyritonavir analog, was also prepared and redocked into CYP3A4. Then the grids were refined as enclosing boxes with length no more than 25 Å by Glide version 6.0 (Schrödinger, LLC, New York, NY, 2013).

Docking simulations were performed using the “Extra Precision” (XP) mode of Glide version 6.0 (Schrödinger, LLC, New York, NY, 2013). The value of Glide Emodel was ranked to determine the best docked pose for multiple conformations [26]. The XP Glide gscores calculated by Glide version 6.0 (Schrödinger, LLC, New York, NY, 2013) of the selected docked poses were used for ranking different ligands [26]. All computations were carried out on a Dell Precision 490n dual processor with Linux OS (Ubuntu 12.04 LTS).

### Statistical analysis

Values are presented as mean ± SD. Differences of the parameters between two groups were analyzed by two tailed student's *t* test. *p* values equal or below 0.05 were considered significant.

## SUPPLEMENTARY FIGURES AND TABLES


