# Supplementary material for: Semi-synthetic ocotillol analogues as selective ABCB1-mediated drug resistance reversal agents
Source: Oncotarget. 2015 Jul 4;6(27):24277–90. doi: 10.18632/oncotarget.4493 (PMC4695185; doi:10.18632/oncotarget.4493)
Supplement: Supplementary file 1 [file oncotarget-06-24277-s001.pdf]

SUPPLEMENTARY FIGURES AND TABLES

Copies of ^{1}H NMR and ^{13}C NMR of **ORA**

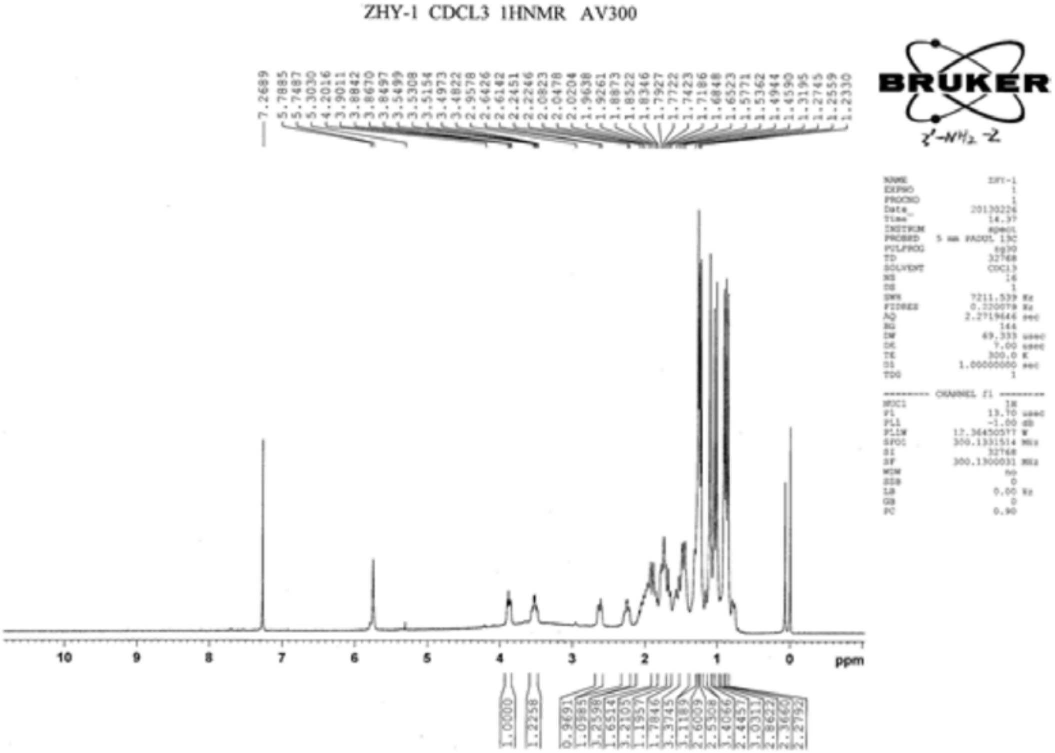

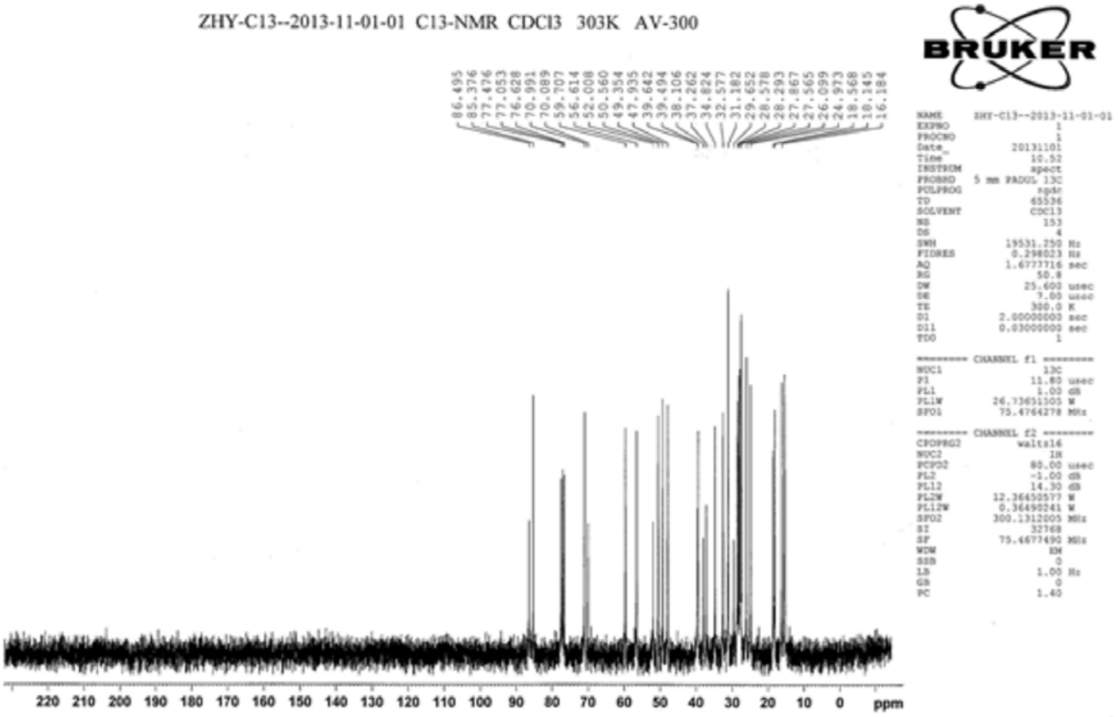

Copies of 1H NMR and 13C NMR of **OSA**

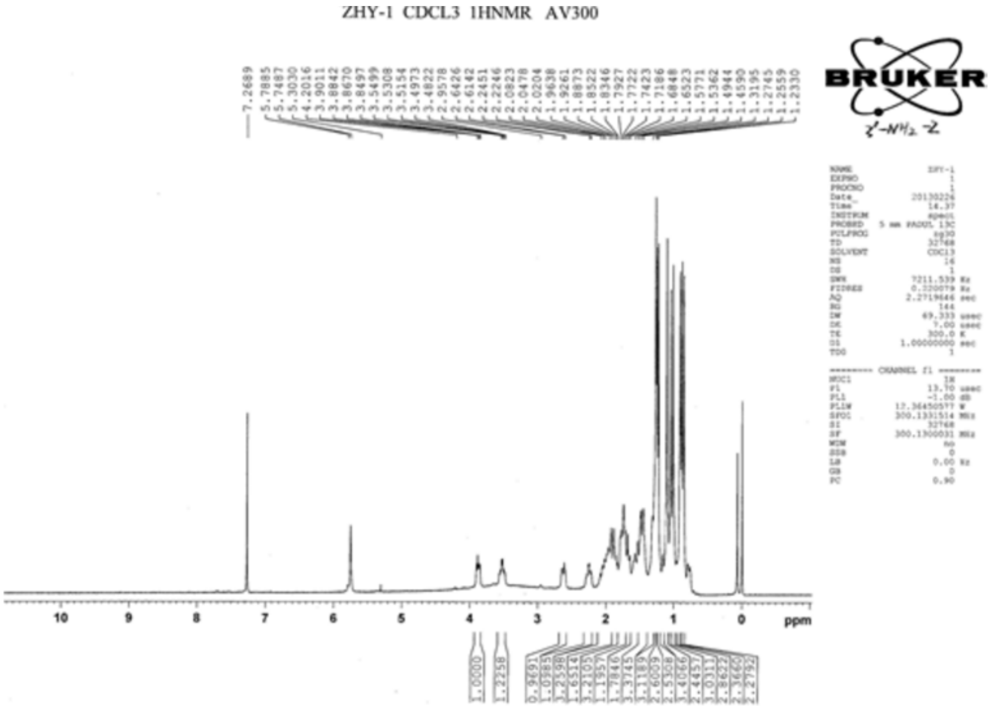

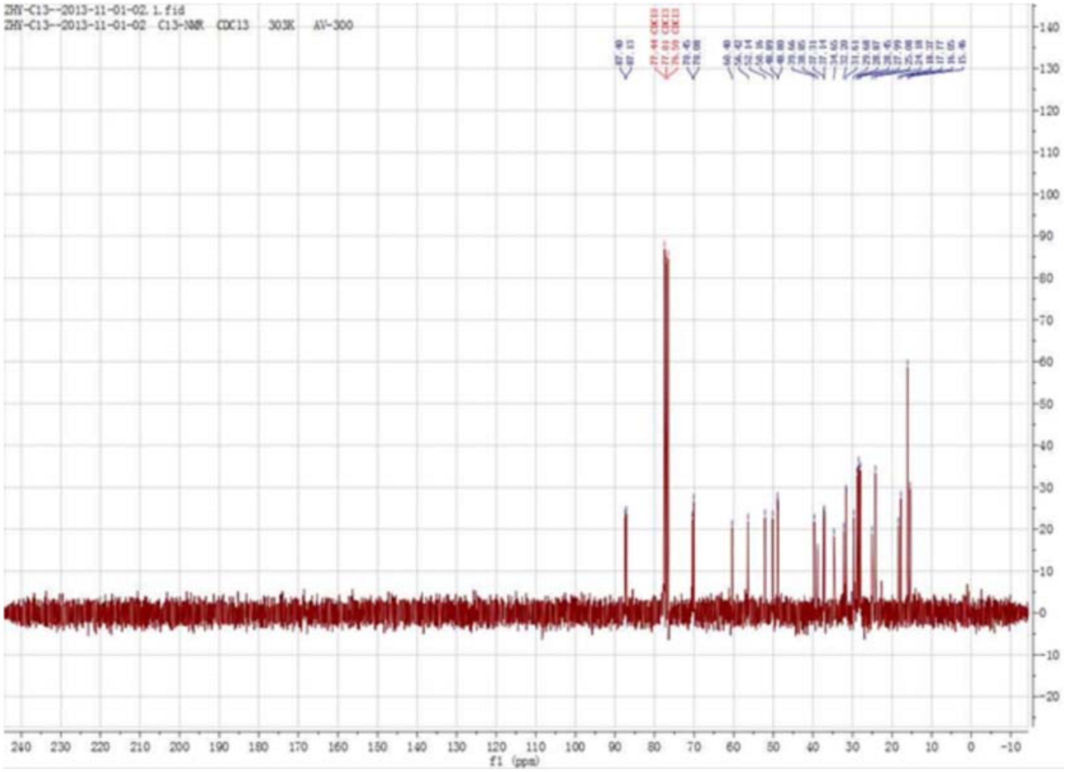

Copies of HRMS of **ORA**

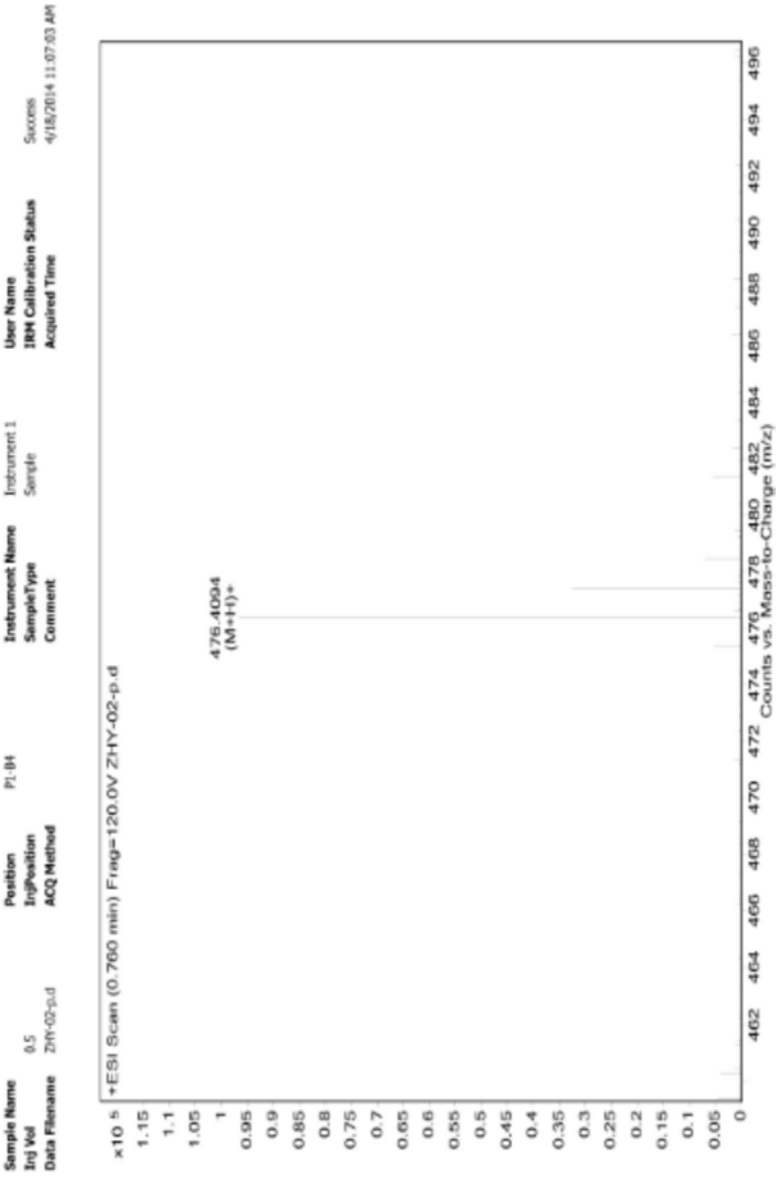

Copies of HRMS of **OSA**

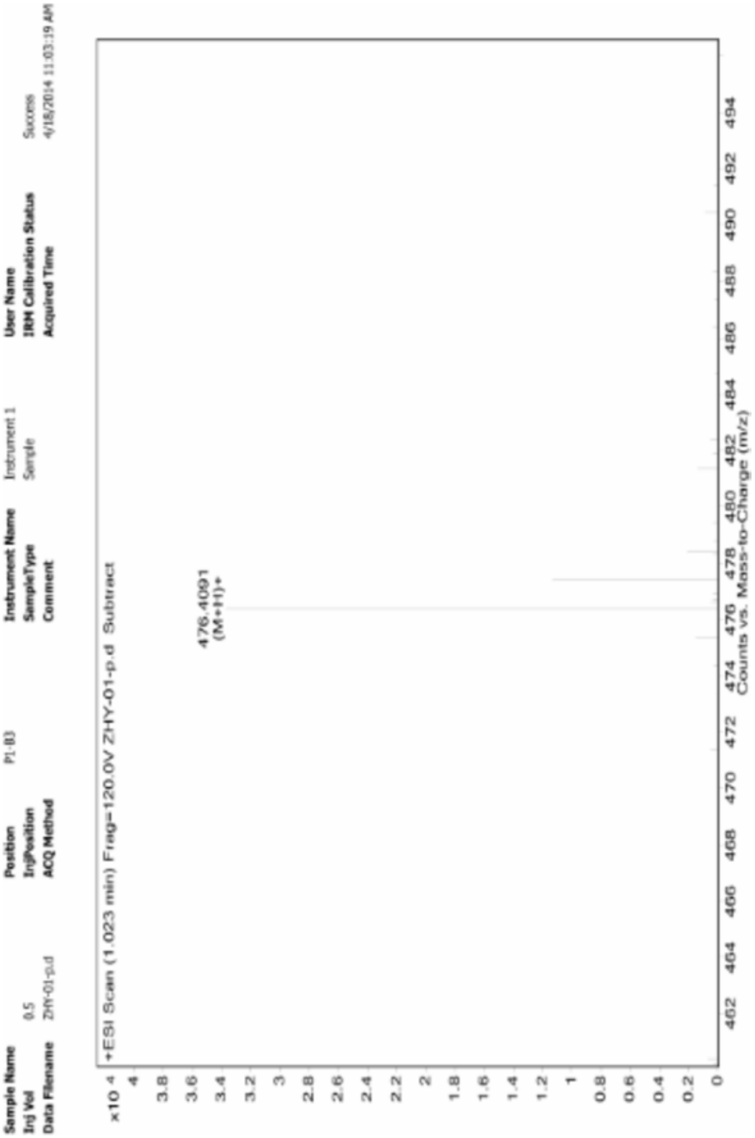

Supplementary Table S1. Sensitivity of cells to substrate drugs with selected aPPD derivatives (5 μM)

Treatment	IC_{50}	Treatment		IC_{50}	
	HEK293	HEK/ABCB1		HEK293	HEK/ABCG2
Paclitaxel	0.18	8.2	Mitoxantrone	0.04	0.92
+PPD	0.19	6.3	+PPD	0.03	0.52
+OR	0.15	7.3	+OR	0.04	0.83
+OS	0.14	7.6	+OS	0.04	0.74
+ORH	0.14	4.9	+ORH	0.03	0.75
+OSH	0.16	5.5	+OSH	0.04	0.71
+ORA	0.14	0.6	+ORA	0.03	0.72
+OSA	0.15	3.1	+OSA	0.04	0.76
+OLA	0.21	8.3	+OLA	0.05	0.82

HEK/ABCG2: HEK293 cells transfected with ABCG2 (wild type) expression vector.
OR/OS: Scaffolds of ocotillol type derivatives; ORH/OSH: 6α-hydroxyl-ocotillol type derivatives; ORA/OSA: 3-amino-ocotillol type derivatives; OLA: Lactone-ocotillol type derivative.

Supplementary Table S2. IC_{15} values of ORA and OSA in selected cell lines

	IC_{15} (μM)	
	ORA	OSA
SW620	3.8	11.2
SW620/Ad300	4.3	8.3
HEK293/pcDNA3.1	4.8	3.2
HEK/ABCB1	5.2	3.6
NCI-H460	13.4	6.4
NCI-H460/MX20	8.6	10.0
HEK/ABCC1	3.7	3.5
